# Trees First Inhibit Then Promote Litter Decomposition in the Subarctic

**DOI:** 10.1111/ele.70063

**Published:** 2025-01-20

**Authors:** Micael Jonsson, Karina E. Clemmensen, Carles Castaño, Thomas C. Parker

**Affiliations:** ^1^ Department of Ecology and Environmental Science Umeå University Umeå Sweden; ^2^ Department of Forest Mycology and Plant Pathology Swedish University of Agricultural Sciences, Uppsala BioCenter Uppsala Sweden; ^3^ Natural Resources Institute Finland (Luke) Helsinki Finland; ^4^ Biological and Environmental Sciences, School of Natural Sciences University of Stirling Stirling UK; ^5^ Ecological Sciences The James Hutton Institute Aberdeen UK

**Keywords:** ectomycorrhizal fungi, ericaceous shrubs, Gadgil effect, leaf litter, mountain birch, priming, root litter, saprophytic fungi, treeline, tundra

## Abstract

Trees affect organic matter decomposition through allocation of recently fixed carbon belowground, but the magnitude and direction of this effect may depend on substrate type and decomposition stage. Here, we followed mass loss, chemical composition and fungal colonisation of leaf and root litters incubated in mountain birch forests over 4 years, in plots where belowground carbon allocation was severed by tree girdling or in control plots. Initially, girdling stimulated leaf and root litter mass loss by 12% and 22%, respectively, suggesting competitive release of saprotrophic decomposition when tree‐mediated competition by ectomycorrhizal fungi was eliminated (Gadgil effect). After 4 years, girdling instead hampered mass loss of root litter by 30%, suggesting late‐stage priming of decomposition in the presence of trees, in parallel with increased growth of shrubs and associated fungi following tree elimination. Hence, different mechanisms driving early‐ and late‐stage litter decomposition should be considered in climate‐feedback evaluations of plant–soil interactions.

## Graphical abstract

Trees may influence organic matter decomposition at the soil surface and in the soil, via effects on the fungal community. However, it is still debated whether such tree effects inhibit (Gadgil effect) or promote (priming) rates of litter decomposition. Here, we show that trees first inhibit and later promote decomposition rates, indicating that, over the long term, priming is a more important mechanism, and that time is critical when performing and evaluating studies on the importance of plant–soil interactions for soil C accumulation.

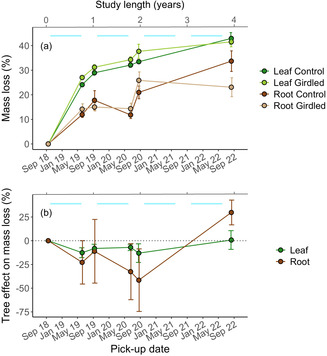

## 1 Introduction

Soils in Arctic and subarctic regions are a globally important carbon (C) stock, which vastly outweighs plant biomass (Köchy, Hiederer, and Freibauer 2015; Post et al. 1982). Since the 1960s, these northern systems have warmed by 1°C–4°C (Hansen et al. 2010), at a rate up to four times faster than the rest of the planet (Chylek et al. 2022; Rantanen et al. 2022). This warming has increased terrestrial plant productivity (Epstein et al. 2012) and has caused treeline advance (Rees et al. 2020) and shrub expansion into previous tundra (Elmendorf et al. 2012; Myers‐Smith et al. 2011; Tape, Sturm, and Racine 2006). In turn, these vegetation responses have increased the aboveground C stock (Epstein et al. 2012), but they may also have modified environmental conditions and processes underlying the soil C stock (Hartley et al. 2012). Such parallel, but potentially asynchronous, changes in above‐ and belowground processes may affect ecosystem C‐sink capacity and feedback to atmospheric CO_2_ concentration and, thus, the global climate (IPCC 2021; Wookey et al. 2009).

To investigate drivers of soil C turnover, most studies have measured plant litter decomposition rates, using leaf litter as the decomposing substrate (Berg and McClaugherty 2014). Leaf litter represents seasonally pulsed inputs of C to the soil surface, and the layer of decomposing leaf litter typically contributes up to 10% of the total soil C stock in arctic and subarctic habitats (Clemmensen et al. 2021). However, plant root litter and necromass of associated mycorrhizal fungi dominate the belowground C input pool and have been found to be the main contributors to long‐term soil C stocks in boreal (Clemmensen et al. 2013; Iversen et al. 2015) and subalpine (Luo et al. 2022) habitats. This is likely true also for arctic and subarctic ecosystems, and especially so due to a generally high plant root:shoot ratio and similar mycorrhizal associations (Mokany, Raison, and Prokushkin 2006). Further, root material generally decomposes more slowly than leaf material (Berg 1984; Herzog et al. 2019; Zhang et al. 2008), facilitating its disproportional contribution to soil organic matter stocks (Kyaschenko et al. 2019). Hence, to accurately predict longer‐term consequences of climate change for the soil C stock, a better understanding of whether drivers of root decomposition differ from those of leaf litter is needed (Clemmensen et al. 2013; See et al. 2019; Sun et al. 2018).

Litter decomposition rate depends on climate (Aerts 1997; Joly, Scherer‐Lorenzen, and Hättenschwiler 2023; Meentemeyer 1978) and litter chemistry, which varies substantially across plant species (Aerts 1997; Heal, Anderson, and Swift 1997; Meentemeyer 1978; Parton et al. 2007), substrate types (Argiroff et al. 2023; Maillard et al. 2023), and decomposition stages (Berg 2014). The decomposition capacity of saprotrophic microbial communities also affects leaf litter (Hobbie and Gough 2004; See et al. 2019), roots (Argiroff et al. 2023) and mycorrhizal mycelia (Fernandez et al. 2016) decay rates. Furthermore, plants provide photosynthate via their roots directly to ECM fungi in exchange for nutrients and water, and some ECM fungi may—especially at later decay stages—participate directly in decomposition of chemically complex organic matter via production of oxidative enzymes (Lindahl et al. 2021; Shah et al. 2016). Both plants and ECM fungi may also release exudates to the wider (mycor)rhizosphere (Vives‐Peris et al. 2019). This source of labile C may stimulate decomposition of more recalcitrant, and potentially older, organic matter by free‐living saprotrophic communities (i.e. ‘priming’; Fontaine et al. 2007; Subke et al. 2011). Hence, increased input of labile C fractions via roots and associated symbionts to soils, following enhanced plant productivity, shrub expansion, or tree advance in the Arctic and subarctic, may result in faster decomposition of soil organic matter, increased CO_2_ efflux, and reduced soil C storage (Clemmensen et al. 2021; Hartley et al. 2012; Parker, Subke, and Wookey 2015; Parker et al. 2021).

ECM fungi can also suppress decomposition activity by saprotrophic fungi (i.e. the ‘Gadgil effect’; Fernandez and Kennedy 2016; Gadgil and Gadgil 1971); elimination of Scots pine roots in boreal forest caused ~10% faster early‐stage leaf litter decomposition, presumably caused by released saprotrophic decomposition (Sterkenburg et al. 2018). Likewise, the elimination of ericaceous shrubs by manual removal of aboveground plants and rhizomes from boreal forest plots increased litter decomposition by ~10% (Fanin et al. 2022; Grau‐Andrés et al. 2020). In nitrogen (N)‐limited boreal and arctic ecosystems dominated by ecto‐ and ericoid mycorrhizal plants, saprotrophic fungi mainly reside in the uppermost litter layer with ample high‐quality organic matter, whereas mycorrhizal fungi dominate in more decomposed organic layers at up to 10 cm depth (Clemmensen et al. 2021; Sterkenburg et al. 2018). The N concentration typically increases with soil depth, and saprotrophic fungi have been found to access N in deeper soil layers and allocate it to support decomposition activities in the litter layer (Frey 2019), particularly if competition by ectomycorrhizal (ECM) fungi is eliminated (Sterkenburg et al. 2018). Although studies typically find support for either priming or a Gadgil effect, the relative strength of ECM‐related stimulation and inhibition could control net C balance of northern forested ecosystems (Mayer et al. 2023). Further, whether fungal communities that decompose leaf and root litter are the same, and whether they display similar competitive mechanisms and respond in similar ways to vegetation shifts, is largely unknown (but see Argiroff et al. 2023). Investigations of these complex above‐ and belowground responses, and interactions between decomposer guilds, are therefore crucial for understanding global C dynamics in subarctic and arctic ecosystems.

We investigated decomposition of leaf litter and fine root litter in a subarctic treeline ecotone over 4 years. To understand the importance of belowground photosynthate allocation by trees, we performed a decomposition experiment in plots with either intact forest or birch trees that had been girdled to reduce photosynthate input to soils (Parker et al. 2020). To disentangle chemical and biotic drivers of variation in mass loss between treatments, between leaf and root litter, and over time, we analysed C:N ratio, litter C‐compound composition and fungal abundance and community composition in the decomposing litters. We hypothesized that (1) leaf litter and root litter have different decomposition trajectories linked to different C‐compound and fungal community composition and dynamics; leaves initially decompose rapidly due to high availability of high‐energy polysaccharides, while roots overall decompose slower due to higher lignin content. We expected saprotrophic Ascomycota to be main decomposers of high‐energy substrates at early stages (Kohout et al. 2018), while saprotrophic Basidiomycota, which can degrade more stable organic matter, would increase over time (Floudas et al. 2012; Kojima et al. 2016). Further, we hypothesized that (2) restricted belowground C allocation with tree girdling leads to larger initial mass loss of leaf litter (i.e. the Gadgil hypothesis) but smaller mass loss of root litter due to lost stimulation of decomposition by root‐mediated C‐inputs (i.e. less priming). Subsequently, we hypothesized that (3) in the long term, trees stimulate rather than inhibit decomposition of both leaf and root litter through stimulated colonisation by ECM fungi.

## 2 Material and Methods

The study was carried out in Swedish Lapland, in the treeline ecotone (Körner and Hoch 2023), with patchy pure stands of mountain birch (*Betula pubescens Ehrh*. ssp. *czerepanovii* (Orlova) Hämet Ahti), which are associated with ECM fungi, 500–600 m above sea level, 5 km southeast of Abisko (68°19′08´´to 68°18′31´´ N and 18°49′00´´to 18°50′24″ E). Mean annual temperature and annual precipitation 2018–2022 were 0.69°C and 269 mm, respectively (Figure S1). On September 9, 2018, leaf and root litter was collected from several stands in the study area. Leaves of mountain birch were collected from the ground beneath several birch individuals and were brought back to the laboratory. To obtain root litter, organic layer material was sieved in the field, and root material was brought back to the laboratory, where fine roots (≤ 2 mm in diameter) were recovered and cleaned in distilled water to remove external organic and inorganic particles. Roots and leaves were each pooled and homogenised and then dried at 40°C for 48 h.

To measure litter mass loss in the field, we used litter bags (10.0 × 6.4 cm, internal dimensions, and 1.0 × 0.1 mm in mesh size). In each litter bag, we placed either 1.0 g of leaf litter or 0.35 g of root litter (± 0.001 g, for both litter types), and then closed the top with a wire. The leaf litter was 100% mountain birch leaves, whereas the root litter consisted of a mixture of the most common vascular plant species in the mountain birch forest at the study area (Parker et al. 2020), that is, 75% of *Empetrum nigrum* *L.* ssp. *hermaphroditum* (Hagerup) Böcher, 15% mountain birch, 7% *Vaccinium vitis‐idaea* *L.* and 3% *V. myrtillus* *L.* (Figure S2a), with proportions estimated from DNA analyses (see below) of pre‐incubation litter. Bulk samples of leaves (4.044 g) and roots (3.904 g) were dried at 60°C for 48 h, and weighed, to determine the dry mass of each litter type. Based on this, the dry mass of litter in each of the litter bags was 0.929 and 0.330 g for leaf and root litter, respectively.

In early June 2017, six paired plots of 20 m diameter were set up in the study area. Each pair consisted of one control (untreated) plot and one plot in which the mountain birches were girdled in mid‐June 2017, to reduce the transfer of photosynthate from the canopy to belowground. For more detailed information on the study site and the plots, see Parker, Subke, and Wookey (2015); Parker et al. (2020). In September 2018, two root litter bags were deployed in the upper organic soil layer (~5 cm below the soil surface), together with two leaf litter bags on top of the organic soil, at four different locations in each plot, making it a total of 16 litter bags. Each of the four litter bags were attached with a metal wire (~5 cm long) to a single, plastic stick stuck into the ground, to ease detection, and a metal clamp was placed over each leaf litter bag to keep it in place and to ensure contact with the organic soil surface. The distance between each set of four litter bags was approximately 5 m, at an equal distance from the centre of the plot. Retrievals of litter bags were made early summer and early autumn over 2 years, that is, June 6 and September 10, 2019, and June 17 and August 20, 2020, corresponding to 9, 12, 21 and 23 months after deployment. At each date, one randomly chosen pair of leaf and root litter bags were retrieved from each plot. At retrieval, the litter bags were brought back to the laboratory, freeze dried, and then stored in a freezer (−20°C). In addition, on August 10, 2022, 47 months after deployment, the remaining litter bags were retrieved from all plots, and one randomly selected pair from each plot was used for further analyses. To determine mass loss, extrinsic organic material and visible ingrown roots (i.e. roots in leaf litter and fresh roots in root litter) were removed from each sample, before the litter was weighed to the nearest 0.001 g. After this, each litter sample was ground to a fine powder in a ball mill, put in a microcentrifuge tube, and stored in a freezer (−20°C), for later analyses (see below).

### 2.1 Analyses of Litter Chemistry and Fungal Abundance and Community Composition

For organic matter quality evaluation, we performed solid state Nuclear Magnetic Resonance (NMR) and C and N analyses of litters before deployment as well as after incubations up to 23 months. Fungal abundance was estimated in all litters based on quantitative real‐time PCR assays of the fungal ITS2 region, and fungal community composition was based on PacBio sequencing of the same marker gene followed by taxonomic and functional assignments by matching to reference sequences in the UNITE database. Our PCR primers also targeted all dominant plant species in our system, and the relative abundance of different plant species in the root mixture before deployment as well as in incubated litters was estimated based on the ITS2 sequencing output. For detailed descriptions of all laboratory methods, see Supporting Information.

### 2.2 Statistical Analyses

To investigate drivers of litter mass loss, we used linear mixed‐effect models (LME), with experimental block (categorical; df = 5) as random factor, to account for the pairwise design (*n* = 6) of the study. We analysed litter mass loss after 9 months (June 2019), with Treatment (i.e. girdling or control) and Litter type (i.e. leaves or roots) as fixed factors. Then, litter mass loss was analysed over the first 23 months (August 2020), with Time (i.e. incubation duration) included as both a fixed and random factor, to account for plots being repeatedly measured. The same analyses were performed for the percentage C and N remaining out of initial C and N mass, respectively, and for the C:N ratio. We also analysed litter mass over the entire 47 months (August 2022), both with Litter type as a fixed factor and for each litter type separately. Finally, we performed an LME on the time‐integrated decay constant (*k*) across all incubations, with Treatment and Litter type as fixed factors and experimental block as a random factor. To calculate *k* for leaf and root litter, we fitted an exponential function to mass remaining at *t* = 0 and at the subsequent five litter‐bag retrievals for each plot and extracted the exponential (*k*) from the obtained function. For all analyses, interactions between fixed factors were removed from final models if *p* > 0.2.

Fungal community composition was analysed by ordinations with CANOCO 5 (Microcomputer Power, Ithaca, NY, USA). Community composition across all samples was visualised by detrended correspondence analysis (DCA). Canonical correspondence analysis (CCA) was used to statistically evaluate the dependence of fungal community composition on Litter type (leaf vs. root, df = 1), Time (i.e. 23 months; continuous, df = 1), Treatment (df = 1), and sequencing depth (continuous, df = 1) across both litter types, and for each litter type separately. To pinpoint the most important independent predictors of community composition, individual factors were included by forward selection (using Holm correction) based on 999 Monte Carlo permutations. Inclusion of predictors was terminated when the model had reached the same level of explained variation as the global test. Experimental block (categorical) was included as a covariate in all analyses. Community data were Hellinger transformed prior to all analyses. We also tested the dependencies of total and guild‐wise fungal abundance, expressed both as relative abundance (out of all fungal reads) and as ITS copy number (per g dried substrate), on Treatment and Time (23 months), in generalised LMEs (‘glmer’), with experimental block as random factor. Lastly, we performed LME on total fungal biomass (total ITS copies), with Treatment and Time (23 months) as fixed factors, and experimental block as random factor, for leaf and root litter (log transformed) separately. All LME (including ‘glmer’) analyses were performed with R (R Core Team 2023), using the package ‘lme4’ (Bates et al. 2015), after visually assessing normality of the data.

## 3 Results

### 3.1 Litter Decomposition

**FIGURE 1:**
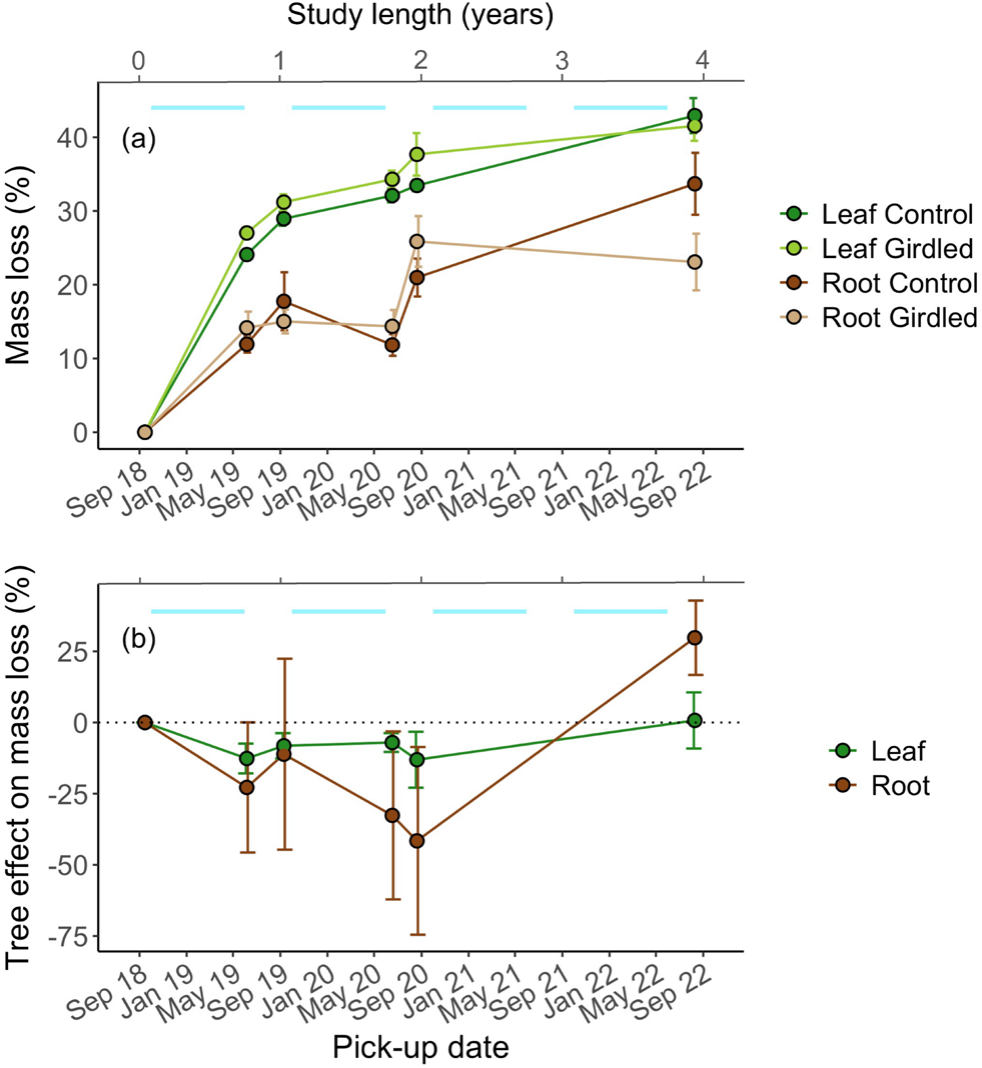
(a) Mass loss (%, not accounting for the block effect), for leaf and root litter, and over time, and (b) Tree effect on litter mass loss (%), that is, percentage difference in mass loss between paired control and girdled plots. For (b) positive values are indicative of priming (a promoting tree effect), whereas negative values are indicative of a Gadgil effect (an inhibitory tree effect). Light blue bands show the periods of snow cover (October–May) in the study area. Error bars represent ±1 standard error of the means.

In the first retrieval after 9 months (June 2019), there was an effect of both Treatment (*t* = 1.98, *p* = 0.048) and Litter type (*t* = −9.61, *p* < 0.001) on litter mass loss (% of initial mass), with a higher mass loss in girdled plots and for leaf litter compared to control plots and root litter, respectively. The interaction Treatment × Litter type was not significant (*p* > 0.2), indicating that girdling resulted in a similar short‐term increase in litter decomposition rates (i.e. a Gadgil effect) for both litter types (Figure 1a,b). On average, this initial Gadgil effect was 12 and 22% for leaf and root litter, respectively, after 9 months. After 23 months (August 2020), there was an effect of Time (*t* = 3.61, *p* < 0.001), and effects of Treatment and Litter type remained unchanged (*t* = 2.28, *p* = 0.022 and *t* = −14.14, *p* < 0.001, respectively). After 47 months (August 2022), the three main factors were still significant (Treatment: *t* = 2.52, *p* = 0.016; Litter type: *t* = −13.84, *p* < 0.001; Time: *t* = 6.80, *p* < 0.001), but now also Treatment × Time was significant (*t* = −2.60, *p* = 0.009), as the treatment effect changed over time (Figure 1a,b); mass loss changed from being higher in girdled plots (i.e. a Gadgil effect) to no difference (leaf litter) or higher in the controls (root litter) at the last pickup (Figure 1b). When analysing litter types separately, this interaction only remained significant for root litter (*t* = −2.18, *p* = 0. 029).

For the decomposition rate *k*, there was an effect of both Treatment (*t* = −2.14, *p* = 0.032) and Litter type (*t* = −3.97, *p* < 0.001), with lower *k* for root litter than for leaf litter, and higher *k* in control plots than in girdled plots, for both root and leaf litter (Figure S3). Thus, late‐stage positive tree effects on litter mass loss (i.e. priming; Figure 1b) was more influential for *k* than were early‐stage Gadgil effects, resulting in continuously increasing priming effects on *k* over time (Figure S3).

### 3.2 Litter Chemical Composition

**FIGURE 2:**
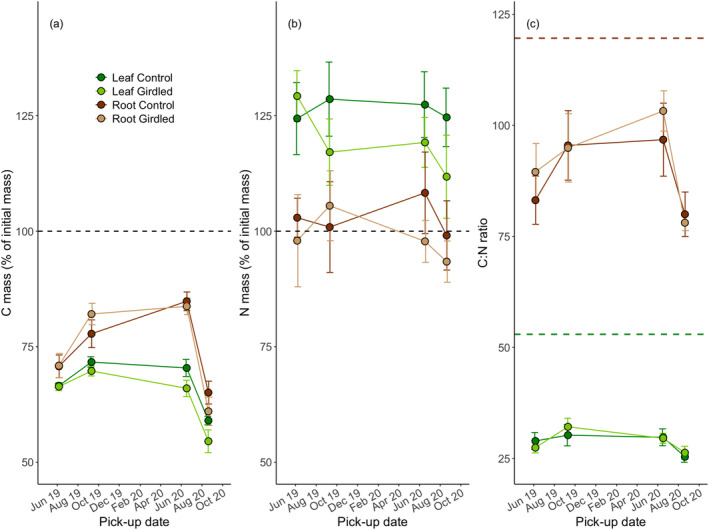
(a) C mass (% of initial C), (b) N mass (% of initial N), and (c) C:N ratio in leaf and root litter in the different treatments over the first 23 months of the study. Dashed lines indicate (a) initial C mass, (b) initial N mass, and (c) initial C:N ratio in root litter (brown) and leaf litter (green). Error bars represent ±1 standard error of the means.

For the first 23 months, there were significant differences in C and N contents (% of initial contents) and C:N ratio between litter types (i.e. *p* always < 0.05; Figure 2). Over the first 9 months (June 2019), leaf and root litter lost ~35% and ~30%, respectively, of initial C mass (Figure 2a). For the next 12 months (until June 2020), root litter gained C although leaf litter C mass remained unchanged, and, after another 9 months, both litter types ended up at ~35%–45% of initial C mass lost. When analysed separately, leaf litter lost more C in girdled plots than in controls (*t* = −2.75, *p* = 0.006), which was not true for root litter (Figure 2a). Leaf litter N initially increased by ~20% and thereafter remained unchanged, whereas root litter N showed no change, over the first 23 months (Figure 2b). There was an initial drop from initial C:N ratio for both litter types, with higher C:N ratios for root litter than for leaf litter, but there was no significant difference in C:N ratio between treatments or across time (Figure 2c).

There were no significant changes over time, or differences between treatments, in C‐compound composition during the first 23 months (Figure S4). However, based on mean relative amounts for the whole 23 months (Figure S5), alkyl‐C tended to increase in leaf litter and decrease in root litter, whereas O alkyl‐C tended to decrease in leaf litter and increase in root litter, compared to initially. Further, in leaf litter, carbonyl‐C tended to increase.

### 3.3 Fungal Community Composition

**FIGURE 3:**
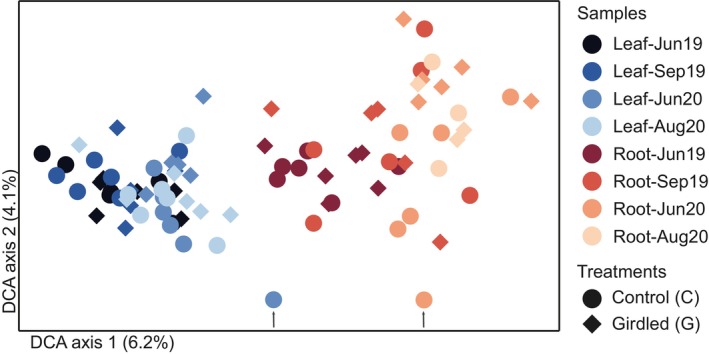
Sample plot from a detrended correspondence analyses (DCA) of total fungal communities in leaf and root litters decomposing over 23 months in experimental plots with intact or girdled subarctic treeline mountain birch forest. Experimental block was a covariate in the analysis. See Table S1 for results from a canonical correspondence analysis.

**FIGURE 4:**
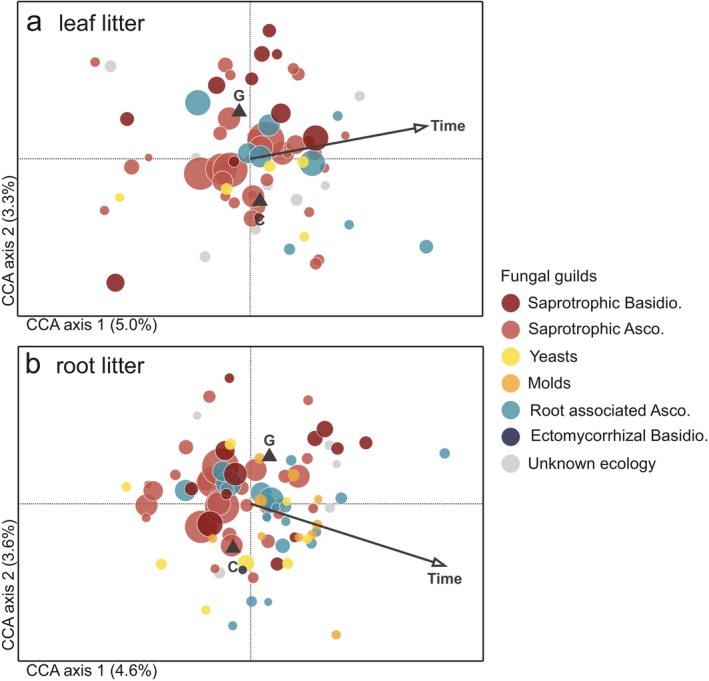
Canonical correspondence analyses of fungal communities in leaf and root litters decomposing over 23 months in experimental plots with intact (C) or girdled (G) subarctic mountain birch canopy in northern Sweden. Canonical correspondence analyses of fungal communities in (a) leaf litter samples and (b) root litter samples, visualising the variation that depend on incubation duration (time) and girdling treatment (G vs. C). Experimental block was a covariate in all analyses. See Table S1 for results from statistical tests.

Fungal communities clearly differed between leaf and root litter and followed different successional trajectories over the first 23 months of incubation (Figure 3). Accordingly, the CCA with forward selection attributed 5.5% of total community variation (after accounting for experimental block) to litter type, 2.2% to incubation duration, and 1.8% to the girdling treatment (*p* < 0.001 for all; Table S1). Fungal communities, also when analysed separately in leaf and root litter, depended on incubation time and the girdling treatment, with about 5% of variation attributed to incubation duration and 3.5% attributed to the girdlig treatment for both litters (Figure 4; Table S1).

**FIGURE 5:**
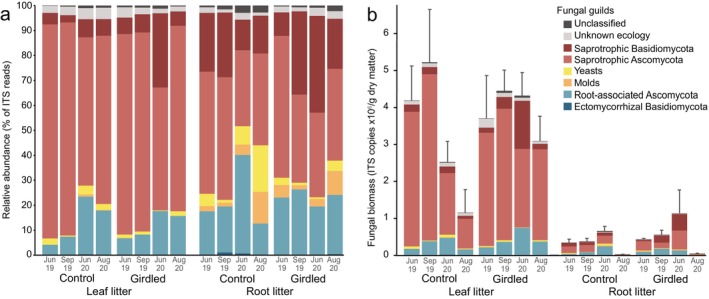
(a) Relative abundance of fungal guilds (out of total fungal communities) and (b) total and guild‐wise biomass estimated by multiplying relative abundances with total fungal ITS copy numbers in leaf and root litter incubated from September 2018 for five periods until August 2020 in control plots and in plots where subarctic mountain birch trees were girdled in 2017. Means (*n* = 3–6). Error bars in (b) represent +1 standard error of the means of total fungal ITS copies as determined by quantitative PCR.

In leaf litter, the summed relative abundance of saprotrophic ascomycetes declined over time, while that of root‐associated ascomycetes increased over time (Figure 5a; Table S2). Girdling further increased the relative abundance of root‐associated ascomycetes in leaf litter, although this effect declined over the 23 months (Treatment × Time interaction; Table S2). In decomposing roots, the relative abundance of moulds and yeasts increased, while that of saprotrophic basidiomycetes overall decreased, with incubation time (Figure 5a; Table S2). Girdling also tended to increase (*p* = 0.058) the relative abundance of saprotrophic basidiomyces in root litter, but the significant interaction with time (*p* = 0.019) indicated that girdling first decreased, then increased, the relative abundance of this guild over the first 23 months (Table S2, Figure 5a). A few ectomycorrhizal fungi (*Polyozellus umbrinus*, *Thelephora terrestris*, *Tomentella* sp.) were found at low relative abundance (< 1%) during the first 23 months of the experiment, but no significant effects of any of the tested factors were found for this guild.

Total fungal biomass (i.e. total number of ITS copies) was substantially higher in leaf litter than in root litter (Figure 5b). In leaf litter, total fungal biomass was higher in girdled plots (*p* = 0.031) and increased over time (*p* < 0.001) but mainly in the girdled plots (i.e. Treatment × Time; *p* = 0.006; Figure 5b). In root litter, total fungal biomass overall increased over time (*p* = 0.039) despite a drastic drop after 23 months, but did not differ between treatments (*p* = 0.647). When guild‐wise relative abundances were multiplied with total fungal ITS copy numbers, estimated absolute abundances of saprotrophic ascomycetes confirmed their decline over time in leaf litter, but this decline was only evident in control plots, whereas this guild stayed abundant in girdled plots throughout the first 2 years. Saprotrophic basidiomycetes peaked in leaf litter incubated in girdled plots at 21 months (June 2020; Figure 5b; Table S2). In root litter, the only observed changes in absolute abundances were overall declines over time in saprotrophic basidiomycetes and saprotrophic ascomycetes.

After 4 years (August 2022), the fungal communities in leaf litter had become further dominated by the saprotrophic basidiomycete guild in both control and girdled plots, while particularly the saprotrophic ascomycetes had declined (Figure S6). In the root litter, girdling had promoted root‐associated ascomycetes, while saprotrophic basidiomycetes had become more abundant in root litter incubated in control plots. Only three ectomycorrhizal basidiomycetes (*Leccinum variicolor, Polyozellus umbrinus*, *Tomentella bryophila* coll.) were found in low abundances (< 1%) in the litters after 4 years (Figure S6). For root litter after 4 years, we observed 15% (control plots) and 25% (girdled plots) ITS‐markers belonging to vascular plants, while no vascular plants were found in the decomposing litters at any of the intermediate incubation durations. This vascular plant composition resembled that of the pre‐incubation root material (~75% of the ITS reads was *E. nigrum* ssp. *hermaphroditum* ; Figure S2).

## 4 Discussion

**FIGURE 6:**
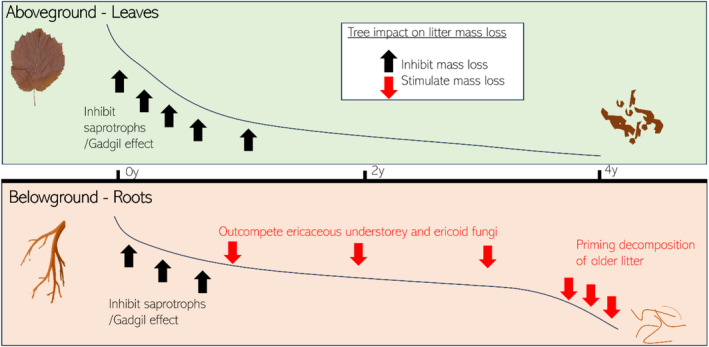
Conceptual graph of litter mass remaining (black lines) indicating how different tree‐mediated effects change over time (red and black arrows), for (a) aboveground leaf litter and (b) belowground root litter. Steepness of line reflects rate of litter mass loss. All processes are likely to occur in parallel but to different degrees depending on the stage of decomposition.

The suppression of saprotrophic decomposition by ECM plants and fungi (i.e. a Gadgil effect) is proposed to have global significance as a mechanism that promotes accumulation of soil organic matter (e.g. Averill, Turner, and Finzi 2014, but see Fernandez and Kennedy 2016; Smith and Wan 2019). Indeed, in agreement with our hypothesis, we found that trees inhibited early stages of litter decomposition, consistent with a Gadgil effect. However, we also found support for our overarching hypothesis that trees over the longer term promote litter decomposition. Hence, after an initial inhibition of litter decomposition, other mechanisms that stimulate litter mass loss became more important (Figure 6). As such, time emerges as a critical factor when performing and evaluating results from studies of tree effects on litter decomposition. As most studies on the importance of above‐ and belowground linkages for litter decomposition have been relatively short term (≤ 2 years), the observed transient Gadgil effect suggests that its importance for long‐term soil C accumulation may be overestimated and therefore needs to be reassessed. Conversely, as highly decomposed organic matter is more stable, promoted litter decomposition by trees may promote long‐term C accumulation, especially for mineral soils (Cotrufo et al. 2019).

The increased decomposition in girdled plots developed during the first autumn, winter and spring, with no additional increase in effect size during the first summer when root and mycorrhizal activity, and thus the potential for competition between ECM and saprotrophic fungi, would be at their highest (Parker et al. 2021). However, the initial 9 months of apparent inhibition of litter saprotrophs by trees occurred when the litters contained the highest amounts of high‐energy polysaccharide compounds (Smith and Wan 2019; Sterkenburg et al. 2018) and litter‐decomposing fungi were most abundant. Interestingly, the tree effect on decomposition rates over the first 23 months was similar for both litter types (Figure 1b), despite differences in fungal community composition, initial litter chemistry and absolute decomposition rates. This may be because roots also contained easily accessible and homogeneous C sources, such as starch and cellulose, as indicated by a high relative abundance of O‐alkyl C (Figure S5), promoting a different saprotrophic community that was also hampered by trees. As such, our results indicate a stabilisation, or initial slowdown, of decomposition rates of the most recent (seasonal) leaf and root litter inputs, which translates into an inhibition of soil C turnover. However, the transient nature of this observed Gadgil effect, especially for root litter, likely reduces its overall importance for total soil C stocks in our study system.

Following the initial Gadgil effect, there was a gradual change over time in mechanisms underlying litter decomposition (Figure 6). First, especially root litter mass loss seemed to be countered by fungal ingrowth, as indicated by increases in C mass (Figure 2), percent O alkyl‐C (Figure S5) and root‐associated fungi (Figure 5). The initial increase in fungal biomass was followed by abrupt decreases in litter C and fungal biomass after 21 months, presumably due to reduced availability of high‐energy compounds in remaining litters. The subsequent transition from a negative (i.e. inhibitory) to no (for leaf litter) or a stimulatory (for root litter) tree effect on litter decomposition, supports a late‐stage priming effect that in the long term may reduce soil C storage (Fontaine et al. 2007; Subke et al. 2011; Hartley et al. 2012). It is particularly interesting that it took more than 23 months for this priming effect to emerge (or become dominant over other mechanisms), given that most studies on litter decomposition are shorter than that. This transition to a priming effect was observed for root litter only, although the tree effect on leaf litter decomposition changed in the same direction. However, importantly, DNA analyses of root litter at termination of the study revealed that some of the apparent priming effect could have been caused by increased fine‐root ingrowth of ericaceous shrubs, rather than reduced litter decomposition rates, in girdled plots compared to in controls. Presumably, remaining root litter mass was so small that newly ingrown biomass of particularly ericoid mycorrhizal *Empetrum* and *Vaccinium* hair roots and associated fungi was enough to counter root litter mass lost through decomposition in the girdled plots compared to in control plots.

Ericaceous shrubs, which often contribute significantly to understory vegetation cover, are theorised to increase soil C stocks through root production and inhibition of decomposition (Clemmensen et al. 2013, 2015; Ward et al. 2022), and their removal has been found to cause loss of belowground C (Fanin et al. 2022; Grau‐Andrés et al. 2020). Accordingly, our results indicate that what looks like late‐stage priming effects (i.e. increased litter decomposition rates) in part may be caused by lower shrub root growth when trees are present. Such competitive advantage of trees over shrubs has previously been observed in these mountain birch forests (Friggens et al. 2023) as well as in boreal Scots pine forests (Mielke et al. 2022). Advancing treelines may therefore cause loss of soil C stocks, not only via priming by ECM fungi (Clemmensen et al. 2021; Parker, Subke, and Wookey 2015; Parker et al. 2021) but also via a reduction in ericaceous shrubs and associated fungi relative to decomposition‐promoting ectomycorrhizal and saprotrophic guilds (Clemmensen et al. 2024).

As hypothesized, leaf and root litter supported different fungal decomposer communities, which resulted in different successional trajectories of colonising communities throughout the study period, and fungal guild composition in leaf litter at later stages of decomposition became more like that in root litter during early stages of decomposition. These results not only support that substrate type (i.e. chemical composition) to a large degree determines decomposer community composition, but also that there is a somewhat predictable (i.e. directional) community succession, such as a general transition from ascomycetes to basidiomycetes becoming the main decomposers at later stages of decomposition (Floudas et al. 2012; Kojima et al. 2016).

We found very little ECM fungal DNA in litter bags, which implies that they had a limited direct role in decomposition of fresh leaf and root litters. However, the shallow organic soils of these subarctic treeline mountain birch forests do contain a range of ECM fungi (Clemmensen et al. 2021; Parker et al. 2022), some with genetic potential to decompose organic matter via oxidative mechanisms (Clemmensen et al. 2021). It is therefore possible that the observed Gadgil effect was linked to ECM fungi that influenced the growth and activity of saprotrophic fungi in the soil, but that this interaction mainly took place outside our litter bags, especially if N needed to support within‐bag litter decomposition mainly exists at deeper soil layers (Frey 2019; Sterkenburg et al. 2018) outside the litter bag.

In this study, we found clear support for an early, transient Gadgil effect caused by competition between saprotrophic fungi and ECM (Sterkenburg et al. 2018). However, we also found a strong, late‐stage priming effect, likely caused by tree root priming when trees were present (control plots) and a greater shrub fine‐root production and associated fungal colonisation when trees were absent (girdled plots). Priming caused by ECM presence and activity has been previously observed in mixed litter layer and humus substrates in these forests (Clemmensen et al. 2021), and we cannot rule out that this mechanism was at work or that it becomes more important at later decomposition stages (after > 4 years) of organic matter decomposition. Hence, what we observed over time in these mountain birch forests was probably the net effect of several underlying mechanisms of soil C turnover acting simultaneously. The prevalence at any given time of any mechanism over the others is likely determined by environmental (i.e. litter type, litter chemistry and soil chemistry, and decomposition stage) influences on competition between saprotrophic and ECM fungi (Mayer et al. 2023; Sterkenburg et al. 2018) and between trees and shrubs (Friggens et al. 2023). For a more comprehensive understanding of above‐ and belowground controls of soil C turnover, potential changes in underlying mechanisms over longer time periods, and differences in mechanisms between soil‐surface and belowground systems, must be considered.

## Author Contributions

M.J. and T.C.P. initiated the project. M.J., T.C.P. and K.E.C. designed the study. M.J. and T.C.P. performed the field experiment, M.J. carried out the NMR analyses, and K.E.C. carried out the fungal community work. C.C. contributed quantitative PCR data. M.J., T.C.P. and K.E.C. analysed the data and wrote the first draft of the manuscript. All authors contributed to data interpretation and revisions of the manuscript.

### Peer Review

The peer review history for this article is available at https://www.webofscience.com/api/gateway/wos/peer‐review/10.1111/ele.70063.

## Supporting information


Data S1.

## Data Availability

Data are openly available at DRYAD (https://doi.org/10.5061/dryad.k6djh9wgn) and code is available at figshare (https://doi.org/10.6084/m9.figshare.26763322).
