# Supplementary material for: Trees First Inhibit Then Promote Litter Decomposition in the Subarctic
Source: Ecol Lett. 2025 Jan 20;28(1):e70063. doi: 10.1111/ele.70063 (PMC11744340; doi:10.1111/ele.70063)
Supplement: Supplementary file 1 — Data S1. [file ELE-28-0-s001.docx]

**Supporting information**

**Material and methods**

*Nuclear Magnetic Resonance (NMR)*

In December 2020, the ground litter samples were prepared for NMR analyses (^13^C Cross-Polarization Magic-Angle Spinning [CP-MAS]), by packing each sample into a 4 mm MAS rotor. The solid state ^13^C NMR spectra were acquired at 125.76 MHz using a Bruker Avance III 500 MHz spectrometer equipped with a 4 mm HX CP-MAS probe and Sample Pro HR-MAS sample changer. The spinning speed was 10 kHz and the CP contact time 1.5 ms with 60 kHz ^13^C spin lock and ramped ^1^H from 45 to 90 kHz was followed by 7 ms acquisition with 1H SPINAL64 decoupling at 83 kHz. For each spectrum 4000 scans were collected with a recovery delay of 2.0 s. The ground litter in each sample was recovered for further analyses (see below), and each rotor was cleaned and re-packed with another litter sample. The NMR analyses were performed continuously over two weeks, starting on December 4.

On December 3, 2020, the chemical shift was calibrated externally to the adamantane CH_2_ signal at 38.48 ppm (Morcombe & Zilm 2003). It should be noted that at the end of the session the magnet had drifted 0.12 ppm (^13^C 15 Hz in 15 days) so the adamantane signal was then at 38.36 ppm. However, as this small drift only corresponds to a shift in less than one point in recorded 4096 points resolution, this was not compensated for in the final data. The spectra were processed with 50 Hz line broadening and automatic zero order baseline correction were applied.

For the results, the spectra were divided and integrated in chemical shift regions with respect to the C compound type (Smernik 2005), i.e. 0–50 ppm (alkyl C), 50–60 ppm (methoxy/N-alkyl C), 60–93 ppm (O-alkyl C), 93–112 ppm (di-O-alkyl C), 112–140 ppm (aromatic), 140–160 ppm (O-aromatic C), and 160–190 ppm (carbonyl C). The integrals were reported on a normalized scale, where the sum of all peaks per spectrum is set to 100.

*C and N analyses*

Dried root and leaf litter material were ground with a ball mill and analysed for C and N content using a Flash Smart^TM^ elemental analyser. The results were used to calculate C and N mass lost as a percentage of initial C and N mass, respectively, and the C:N ratio, over the first 23 months.

*Fungal abundance*

DNA was extracted from 50 mg of milled material using the NucleoSpin Soil kit (Macherey-Nagel, Düren, Germany). The DNA concentration was quantified spectrophotometrically (Thermo Scientific, Wilmington, DEand extracts diluted 1:5. PCR inhibition was checked in all the samples by qPCR using SYBR Green on a BioRad CFX Connect Real-Time system, by amplifying known amounts of pGEM-16S plasmid (Methods S1). Specifically, We checked inhibition in DNA samples by amplifying 1×105 copy numbers of pGEM-16S plasmid (Promega, WI, USA) spiked into qPCR reactions containing either the DNA extracts or sterile water. Linearized plasmids were amplified with plasmid-specific primers (M13F and M13R) in a 20 µl reaction. No inhibition was detected. Fungal abundance in the samples was estimated by quantification of the fungal internal transcribed spacer region 2 (ITS2) using the primers fITS7 (Ihrmark *et al*. 2012), ITS4 (White *et al*. 1990) and ITS4arch (Sterkenburg *et al*. 2015) in a 20 µl reaction, with primers at 0.5 μM (fITS7), 0.3 μM (ITS4) and 0.15 μM (ITS4arch), BSA (0.1%), iQ-SYBR Green (1x), and 2 μL of template. PCR conditions were an initial denaturation at 95 ℃ for 5 min, followed by 40 cycles of denaturation at 95 ℃ for 30 s, annealing at 57 ℃ for 30 s, synthesis at 72 ℃ for 40 s and 78 ℃ for 5 s. Serial dilutions of known amounts of linearized plasmid containing the ITS2 fragment were used as standard. Samples were run in duplicate in two distinct plates, but results were repeated if coefficient of variation between duplicates was > 30%.

*Fungal communities*

Fungal community ITS2 amplicons for sequencing were generated by PCR using the same primers as above, but with primers elongated with 8 base long sample-identification tags (Clemmensen *et al*. 2023). PCRs were run in technical duplicates on x20 diluted template DNA and amplification cycles minimized to obtain unbiased amplification of ITS2 regions of different lengths (Castaño *et al.* 2020). The PCR mix consisted of 0.2 mM dNTPs, 0.75 mM MgCl_2_, 0.5 µM of fITS7 primer, 0.3 µM of ITS4 primer, 0.1µM of ITS4arch primer and 1.25 U of DNA polymerase (Dream Taq, Fermentas, Sweden) in 50 µl reactions. Thermal cycling conditions were 5 min at 95 °C, followed by 27-35 cycles of 30 sec at 95 °C, 30 sec at 56 °C and 30 sec at 72 °C, and a final 7 min at 72 °C. Amplicons were purified using the Agencourt AMPure kit (Beckman Coulter, Beverly, MA, USA) and concentrations measured fluorometrically (Qubit high sensitivity kit, Invitrogen, Carlsbad, CA, USA). Amplicons from the first 4 harvests were mixed in equal amounts into one sequencing pool, and amplicons from the last harvest were mixed into a separate pool. Both pools were further cleaned with the Cycle Pure kit (EZNA, Omega Bio-Tek, Nocross, GA, USA) and their amplicon size distribution checked (Agilent Bioanalyzer, Santa Clara, CA, USA). Adaptor ligation and Pacific Biosciences sequencing were performed at Uppsala Genome Centre (SciLifeLab); the first pool was sequenced on a Sequel I SMRT cell and the second pool on a Sequel II SMRT cell. This difference in sequencing platform may have caused a batch effect on community composition, and therefore data from the last harvest is given as supplementary data.

Raw sequences were quality filtered and clustered using the bioinformatics pipeline SCATA (http://scata.mykopat.slu.se/; Ihrmark *et al.* 2012). Quality control required an average read score of >20 and a score of >3 for individual bases, 100% match with the sample-identification tags and at least 90% match with the primer sequences. After removing reads only occurring once, high quality reads were clustered into species level clusters by single-linkage clustering using a 98.5% sequence similarity criterion for pairwise comparisons (Usearch; Edgar 2010). Due to the different sequencing technologies, the clustering was done separately for the two pools, resulting in a total of 190,154 high quality sequences assembled into 722 global clusters for the first pool (91 samples), and 218,459 sequences assembled into 965 clusters for the second pool (23 samples). Cluster occurrences with less than 4 (pool 1) or 15 (pool 2) reads in an individual sample were set to zero to remove potential erroneous sample assignments (Carlsen *et al*. 2012). The most abundant 208 (pool 1) and 200 (pool 2) clusters summing up to at least 95% of total amplicons in each pool were taxonomically annotated by 1) including all fungal species hypotheses reference sequences in the UNITE database (https://unite.ut.ee/; Abarenkov *et al.* 2010) in the clustering process, and 2) further assessment for taxonomic identity and functional guild, by comparing reference sequences to all global species hypotheses using massBLASTer in the UNITE PlutoF module.

After removing non-fungal clusters (pool 1: 4 clusters, 661 reads; pool 2: 30 clusters, 21,272 reads), the final data sets consisted of 342 fungal species (103,129 reads; pool 1) and 334 fungal species (139,369 reads; pool 2). The fungal species occurrences were aligned between the two pools by including representative sequences from clusters in pool 1 as a reference set when clustering pool 2 (54% overlap in species). The following fungal guilds were classified: ectomycorrhizal fungi (mainly Basidiomycota), other root associated fungi (mainly Ascomycota; including ericoid mycorrhizal species) and saprotrophic fungi with subgroups of saprotrophic Ascomycota, Basidiomycota, molds and yeasts.

The proportion ITS2 markers belonging to vascular plants (*Betula* spp., *Empetrum nigrum, Vaccinium vitis-idaea* and *V. myrtillus*, together representing 100 and 73% of non-fungal reads in the two pools, respectively) out of total quality filtered ITS2 amplicons was used as a proxy for root ingrowth into the bags.

Representative sequences, classifications and data matrices are available at DRYAD (https://datadryad.org/stash/share/cMgVT9v-b_4kJ2_OeGPvQ_lhuBda6MVQvs-eXio45kI). Sequence raw data are archived at the Sequence Read Archive (www.ncbi.nlm.nih.gov/sra) under accession number PRJNA1101716.

**Figures**


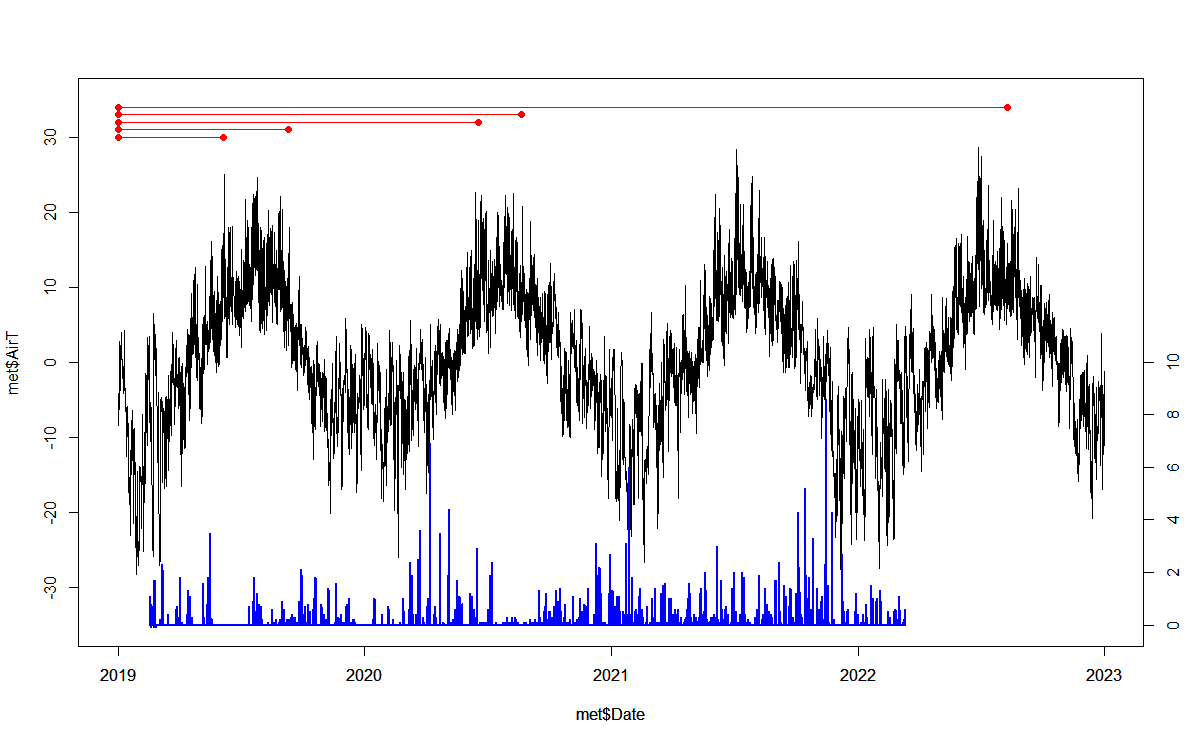


**Figure S1** Air temperature (black) and precipitation (blue) from the Abisko weather station, as well as the time period for each litter harvest (red lines).

**
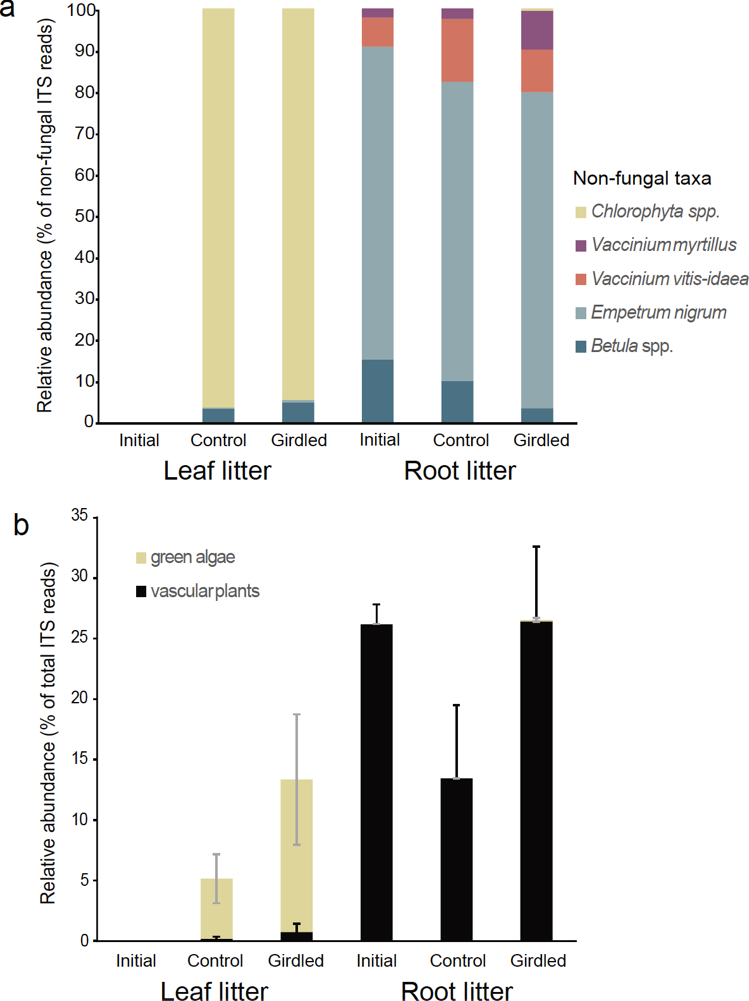
**

**Figure S2** Proportions of ITS2 markers assigned to vascular and nonvascular plant species out of (a) all non-fungal sequences and (b) total quality filtered amplicons (i.e. all plants and fungi), as indicators of presence of roots of vascular plants (*Betula* spp., *Empetrum nigrum, Vaccinium vitis-idaea* and *V. myrtillus*) and algae (*Chlorophyta* spp.) in the pre-incubation (“initial”) root substrates and leaf and root substrates after 4 years of field incubation in control and girdled birch forest plots. No non-fungal markers were captured in substrates incubated at the intermediate incubation durations. Pre-incubation leaf samples yielded insufficient DNA. Error bars represent + 1 standard error of the means (*n* = 3-6).


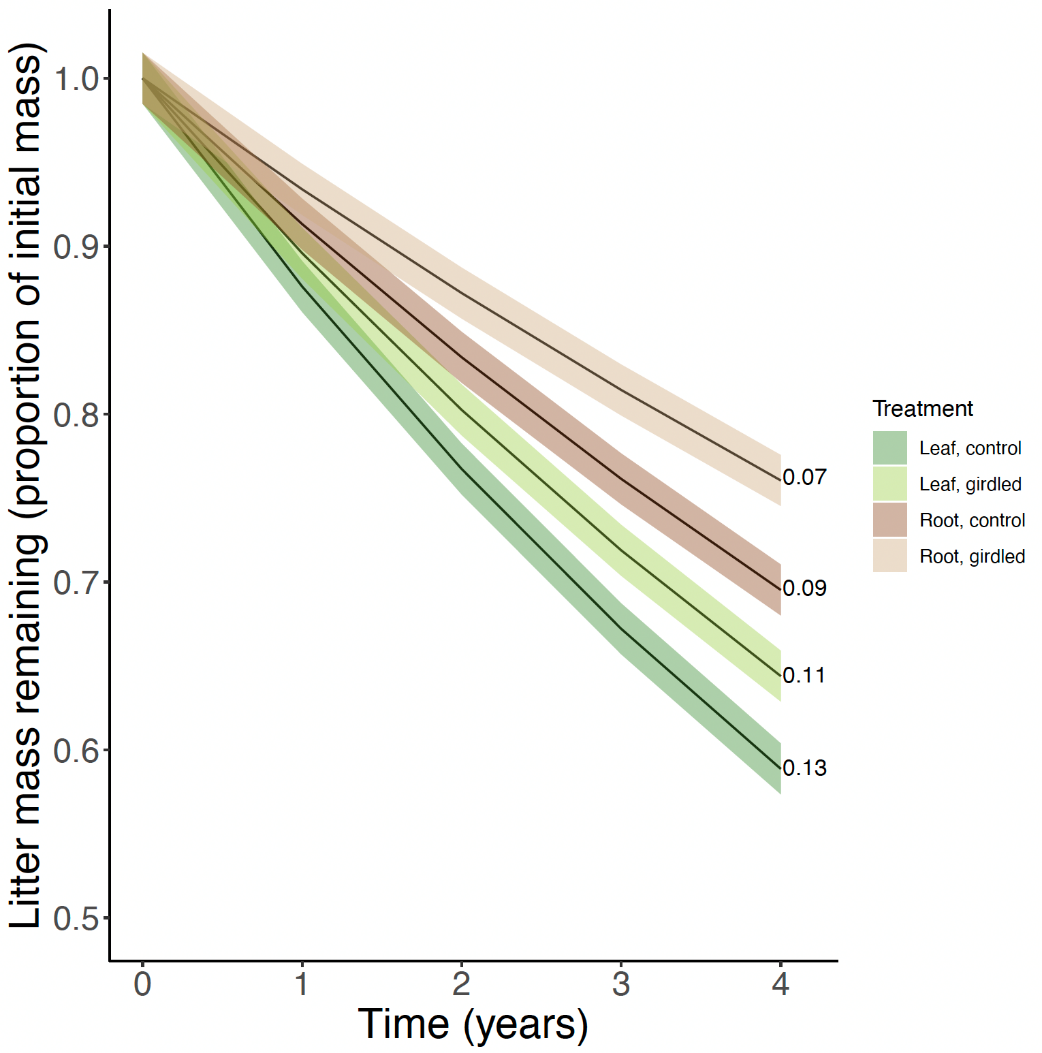


**Figure S3** Predicted litter mass remaining, based on a linear mixed-effect model on *k*, for leaf and root litter in control or girdled plots over 4 years. Small numbers are the estimated *k* values, based on measured mass loss in all plots. Coloured bands around mean represent 95% confidence intervals.


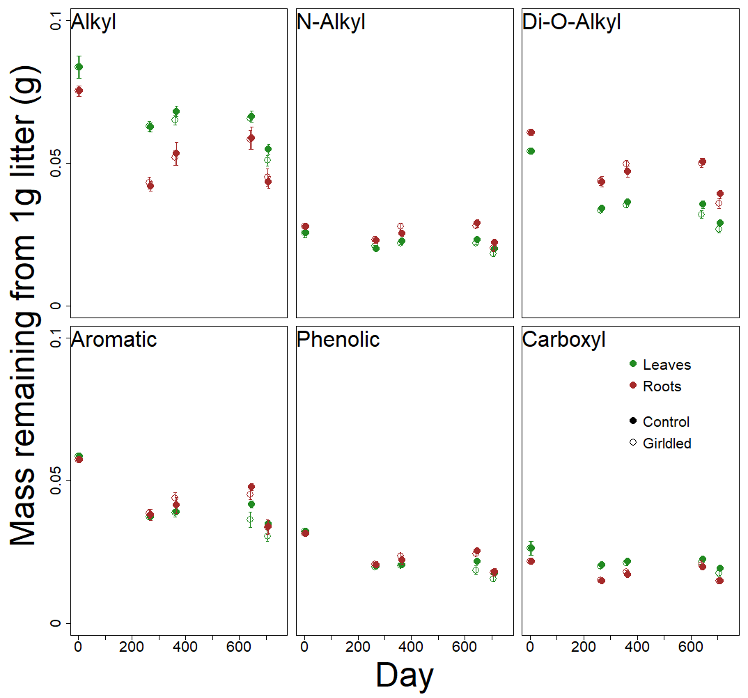


**Figure S4** Remaining mass of leaf and root litter C from 6 major chemical shift regions in girdled and control forest plots over the first 23 months of decomposition.


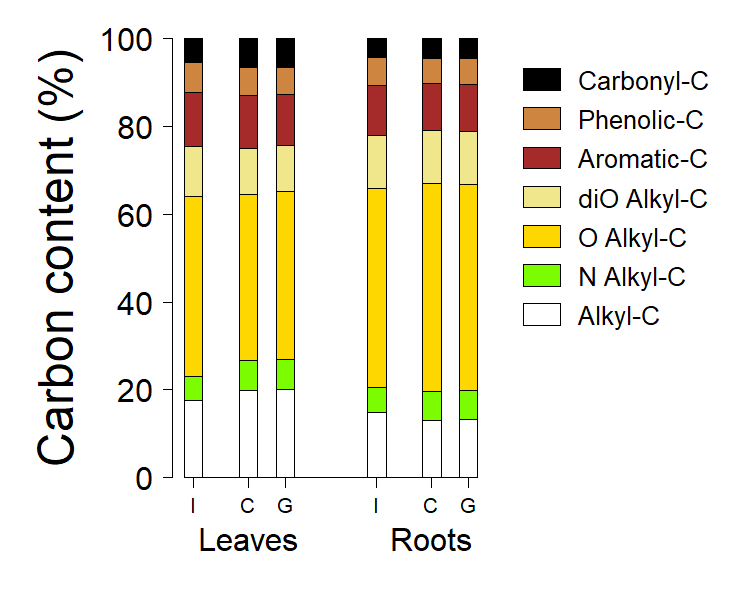


**Figure S5** Relative amounts of leaf and root litter C from 6 major chemical shift regions in girdled (G) and control (C) plots, and in initial litter (I). There were no significant changes in C-compound composition over time (Fig. S4) so only initial values and mean values for the first 23 months are presented.

**Figure S6** Relative abundance of fungal guilds based on ITS metabarcoding in leaf and root litter incubated from September 2018 for five periods until August 2022 in control plots and in plots where the canopy birch trees were girdled in 2017. Pre-incubation (“Initial”) leaf samples yielded insufficient DNA. Means (*n* = 3-6).

**Tables**

**Table S1** Canonical correspondence analyses (CCAs) of fungal communities in leaf and root litters decomposing over two years in experimental plots with intact or girdled subarctic mountain birch canopy in northern Sweden. CCAs were run separately for total fungal communities in (a) all litter, (b) leaf litter, and (c) root litter samples. The effects of litter type (df = 1; only in a), incubation time (df = 1), girdling treatment (df = 1) and sequencing depth (df = 1) were tested by 9999 Monte Carlo permutations in global models including all predictors and in models based on forward selection of the explanatory variables. Experimental block (df = 5) was a covariate in all analyses. Sequencing depth was not contributing significantly to variation in community composition and not included in the forward selection models. Percentage variation explained out of total community variation is given for each explanatory factor and for all included factors. *adj. = adjusted by Holm correction for multiple comparisons.

1. CCA of all fungi (330) across all litter samples (*n* = 87):

| Main effect | *F* (explained variation) | *P* (adj.)* |
| --- | --- | --- |
| *Global test including all factors:* |  |  |
| First axis | 4.8 | 0.0001 |
| All axes | 2.3 | 0.0001 |
| Sum of partial variation: | 7.96 |  |
| **Explained variation (adj.):** | **10.6 (6.0)%** |  |
|  |  |  |
| *Selection of explanatory factors:* |  |  |
| Litter type | 4.7 (5.5%) | 0.0006 |
| Time | 1.9 (2.2%) | 0.0006 |
| Girdling | 1.5 (1.8%) | 0.0006 |
| Sum of partial variation: | 7.96 |  |
| **Explained variation (adj.):** | **9.5 (6.0)%** |  |

1. CCA of all fungi (193) in leaf litter samples (*n* = 46):

| Main effect | *F* (explained variation) | *P* (adj.)* |
| --- | --- | --- |
| *Global test including all factors:* |  |  |
| First axis | 2.0 | 0.0001 |
| All axes | 1.5 | 0.0001 |
| Sum of partial variation: | 4.16 |  |
| **Explained variation (adj.):** | **10.7 (3.5)%** |  |
|  |  |  |
| *Selection of explanatory factors:* |  | |
| Time | 2.0 (4.9%) | 0.0004 |
| Girdling | 1.4 (3.3%) | 0.007 |
| Sum of partial variation: | 4.16 |  |
| **Explained variation (adj.):** | **8.3 (3.4)%** |  |

1. CCA of all fungi (241) in root litter samples (*n* = 41):

| Main effect | *F* (explained variation) | *P* (adj.)* |
| --- | --- | --- |
| *Global test including all factors:* |  |  |
| First axis | 1.5 | 0.02 |
| All axes | 1.3 | 0.003 |
| Sum of partial variation: | 5.08 |  |
| **Explained variation (adj.):** | **10.6 (2.3)%** |  |
|  |  |  |
| *Selection of explanatory factors:* |  | |
| Time | 1.6 (4.4%) | 0.002 |
| Girdling | 1.3 (3.7%) | 0.04 |
| Sum of partial variation: | 5.08 |  |
| **Explained variation (adj.):** | **8.1 (2.5)%** |  |

| **Relative abundance** | | | | | | | | | | |
| --- | --- | --- | --- | --- | --- | --- | --- | --- | --- | --- |
|  | ***Root associated*** | | ***Mold*** | | ***Yeast*** | | ***SAPa*** | | ***SAPb*** | |
|  | *z* | *P* | *z* | *P* | *z* | *P* | *z* | *P* | *z* | *P* |
| ***Leaves*** |  |  |  |  |  |  |  |  |  |  |
| Treatment | 2.136 | **0.033** |  |  | 0.571 | 0.568 | -0.494 | 0.622 | 0.863 | 0.388 |
| Time | 7.719 | **<0.001** |  |  | 0.974 | 0.330 | -2.269 | **0.023** | 1.185 | 0.236 |
| Treatment 🞨 Time | -2.102 | **0.036** |  |  | -1.040 | 0.298 | 0.287 | 0.774 | -0.182 | 0.856 |
|  |  |  |  |  |  |  |  |  |  |  |
| ***Roots*** |  |  |  |  |  |  |  |  |  |  |
| Treatment | 0.658 | 0.511 | 1.155 | 0.248 | 0.635 | 0.526 | -0.071 | 0.943 | -1.893 | ***0.058*** |
| Time | 0.353 | 0.724 | 2.639 | **0.008** | 1.999 | **0.046** | -1.340 | 0.180 | -1.919 | ***0.055*** |
| Treatment 🞨 Time | -0.377 | 0.706 | -1.135 | 0.256 | -1.653 | ***0.098*** | -0.098 | 0.922 | 2.336 | **0.019** |
|  |  |  |  |  |  |  |  |  |  |  |
| **ITS copy numbers** | | | | | | | | | | |
| ***Leaves*** |  |  |  |  |  |  |  |  |  |  |
| Treatment | -0.479 | 0.632 |  |  | 0.071 | 0.943 | -2.091 | **0.036** | -0.932 | 0.352 |
| Time | 0.000 | 1.000 |  |  | -0.048 | 0.962 | -5.482 | **<0.001** | -0.998 | 0.318 |
| Treatment 🞨 Time | 0.970 | 0.332 |  |  | -0.345 | 0.730 | 2.720 | **0.007** | 1.725 | ***0.085*** |
|  |  |  |  |  |  |  |  |  |  |  |
| ***Roots*** |  |  |  |  |  |  |  |  |  |  |
| Treatment | 0.073 | 0.942 | 0.328 | 0.743 | 0.908 | 0.364 | -0.140 | 0.889 | -0.799 | 0.424 |
| Time | -0.351 | 0.725 | -0.316 | 0.752 | 0.311 | 0.756 | -1.783 | ***0.075*** | -2.172 | **0.030** |
| Treatment 🞨 Time | -0.009 | 0.993 | -0.252 | 0.801 | -1.688 | ***0.091*** | 0.275 | 0.783 | 1.101 | 0.271 |

**Table S2** Results from generalised linear models models on fungal guilds between girdling and control treatments and over the first 23 months of the study, for leaf and root litter separately, and for both relative abundance and number of ITS copies of different fungal guilds. Bold numbers indicate significance at *P* < 0.05 and bold italics indicate marginal significance (*P* < 0.1). Root associated = root-associated ascomycetes, SAPa = saprotrophic ascomycetes, SABb = saprotrophic basidiomycetes.

**References**

Abarenkov, K., Nilsson, R.H., Larsson, K.-H., Alexander, I.J., Eberhardt, U., Erland, S., *et al*. (2010). The UNITE database for molecular identification of fungi--recent updates and future perspectives. *New Phytol*., 186, 281–285.

Carlsen, T., Aas, A.B., Lindner, D., Vrålstad, T., Schumacher, T. & Kauserud, H. (2012). Don't make a mista(g)ke: is tag switching an overlooked source of error in amplicon pyrosequencing studies? *Fungal Ecol.*, 5, 747–749.

Castaño, C., Berlin, A., Brandström Durling, M., Ihrmark, K., Lindahl, B.D., Stenlid, J., *et al*. (2020). Optimized metabarcoding with Pacific biosciences enables semi-quantitative analysis of fungal communities. *New Phytol.,* 228, 1149–1158.

Clemmensen, K.E., Ihrmark, K., Brandström Durling, M. & Lindahl, B.D. (2023). Sample preparation for fungal community analysis by high-throughput sequencing of barcode amplicons. In: Martin, F. & Uroz, S. (eds), *Microbial Environmental Genomics.* Springer, pp. 37–64.

Edgar, R.C. (2010). Search and clustering orders of magnitude faster than BLAST. *Bioinformatics*, 26, 2460–2461.

Ihrmark, K., Bödeker, I.T.M., Cruz-Martinez, K., Friberg, H., Kubartova, A., Schenck, J., *et al.* (2012). New primers to amplify the fungal ITS2 region - evaluation by 454-sequencing of artificial and natural communities. *FEMS Microbiol. Ecol*., 82, 666–677.

Morcombe, C.R. & Zilm, K.W. (2003). Chemical shift referencing in MAS solid state NMR. *J. Magn. Reson*., 162, 479–486.

Smernik, R.J. (2005). Solid-state 13C NMR spectroscopic studies of soil organic matter at two magnetic field strengths. *Geoderma*, 125, 249–271.

Sterkenburg, E., Bahr, A., Brandström Durling, M., Clemmensen, K.E. & Lindahl, B.D. (2015). Changes in fungal communities along a boreal forest soil fertility gradient. *New Phytol*., 207, 1145–1158.
